# Successful closure of large mucosal defect with novel anchor-pronged clip after gastric endoscopic submucosal dissection

**DOI:** 10.1055/a-2334-1103

**Published:** 2024-06-25

**Authors:** Kenichiro Okimoto, Tomoaki Matsumura, Keisuke Matsusaka, Yuki Ohta, Takashi Taida, Jun Kato, Naoya Kato

**Affiliations:** 1 Department of Gastroenterology, Graduate School of Medicine, Chiba University, Chiba, Japan; 2 92154 Department of Pathology, Chiba University Hospital, Chiba, Japan; 3 92154 Endoscopy Center, Chiba University Hospital, Chiba, Japan

Delayed bleeding after gastric endoscopic submucosal dissection (ESD) is a major complication 1 that sometimes leads to shock, and mucosal defect closure to prevent delayed bleeding was reported 2 . However, the thickness of the gastric mucosa and proper muscle layer makes suturing difficult 3 . A recent study reported the closure of an intermediate-size mucosal defect post-gastric ESD with a novel anchor-pronged clip (MANTIS Clip; Boston Scientific, Marlborough, Massachusetts, USA) ( Fig. 1 ) 4 . Here, we present the successful closure of large mucosal defect with a MANTIS Clip after gastric ESD ( Video 1 ).

**Fig. 1:**
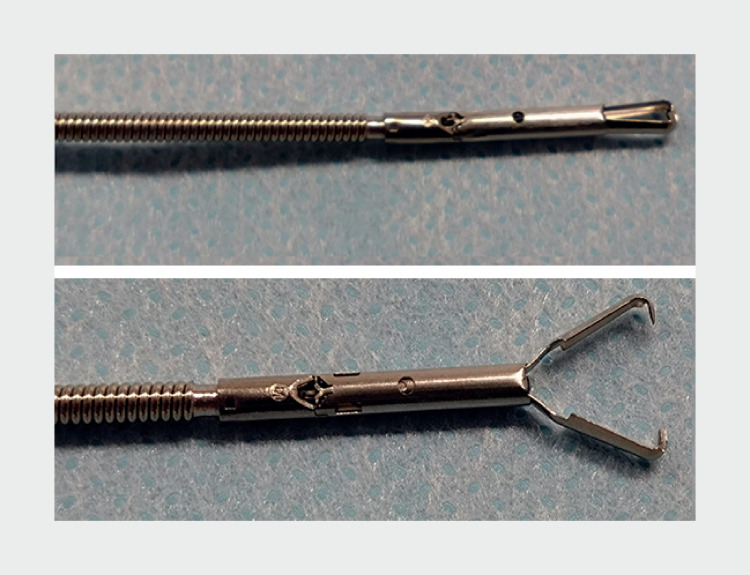
Novel anchor-pronged clip. The clip is re-openable and rotational.

Successful closure of large mucosal defect after gastric endoscopic submucosal dissection.Video 1

The case involved a woman in her 90s on continued low-dose aspirin therapy. A 40-mm lesion was identified on the posterior wall of the lower body in the stomach ( Fig. 2 **a** ). ESD was performed for en bloc resection, resulting in a mucosal defect approximately 60 mm in size ( Fig. 2 **b** ). The pathological finding was intramucosal carcinoma with a negative horizontal and vertical margin. The defect was closed using a similar approach as a mucosa-submucosa closure after colorectal ESD 5 . First, one side of the mucosa was grasped with the MANTIS Clip. The clip was then moved to the opposite muscle layer, opened once, and after sufficient air aspiration, the clip was used to grasp the muscle layer ( Fig. 2 **c, d** ; Fig. 3 **a, c** ). After sufficient shrinkage of the mucosal defect, complete closure without obvious dead space was achieved by adding mucosa-mucosa and mucosa-muscle closure with normal clips (16-mm Sure Clip; Micro-Tech Co., Ltd, Nanjing, China) or EZ Clip (Olympus Medical Systems, Tokyo, Japan) ( Fig. 2 **e** ; Fig. 3 **d–g** ). In total, three MANTIS Clips, six Sure Clips, and four EZ Clips were applied. The closure time was 14 minutes. The patient was discharged according to schedule without experiencing delayed bleeding.

**Fig. 2:**
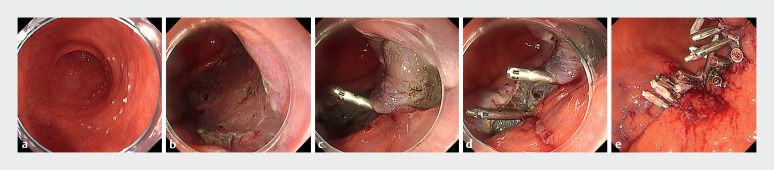
Closure of a large mucosal defect after gastric endoscopic submucosal dissection (ESD). **a** The lesion was diagnosed endoscopically as a 40-mm diameter IIc. Markings were placed around it. **b** The mucosal defect after ESD. The diameter was estimated to be around 60 mm. **c** The mucosal defect was shrunk with a MANTIS Clip (mucosa-muscle closure was performed). **d** The mucosal defect was further shrunk using the same method. **e** Complete closure was achieved by adding mucosa-mucosa and mucosa-muscle closure with normal clips.

**Fig. 3:**
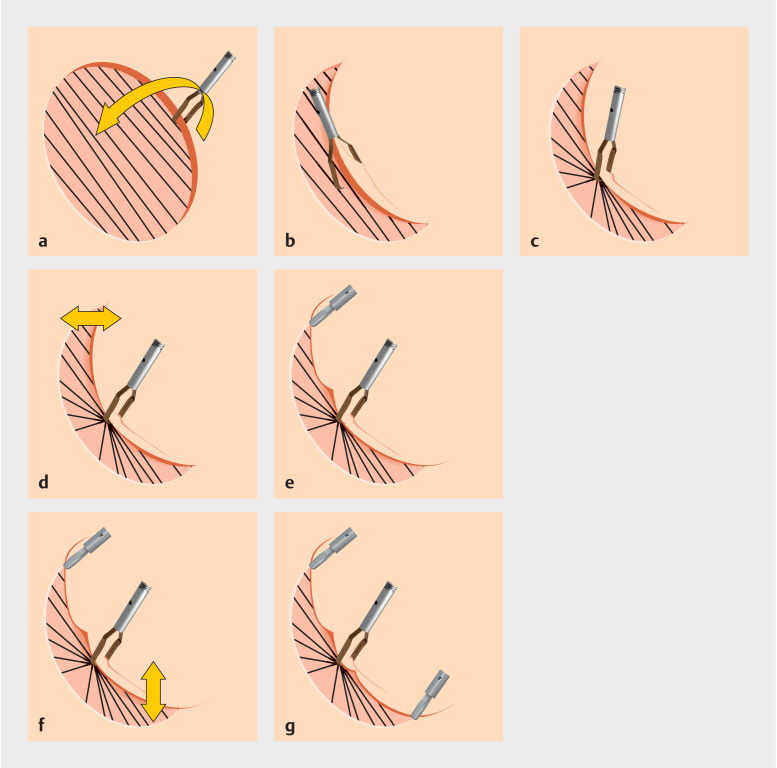
Closing method schema. The black diagonal lines represent the course of muscle fibers. **a–c** When the mucosal defect is large after ESD, grasp one side of the mucosa with a MANTIS Clip. Then move the clip to the opposite muscle layer, open the clip once, and after sufficient air aspiration, grasp the muscle layer. **d, e** When the mucosal defect is small, mucosa-mucosa closure after sufficient air aspiration is performed with a normal clip. **f, g** When the mucosal defect is intermediate in size, mucosa-muscle closure after sufficient air aspiration is performed with a normal clip.

The novel anchor-pronged clip was thought to be useful in closing even large mucosal defects after gastric ESD.

Endoscopy_UCTN_Code_TTT_1AO_2AO

## Notes

Endoscopy E-Videos https://eref.thieme.de/e-videos
*E-Videos* is an open access online section of the journal *Endoscopy* , reporting on interesting cases and new techniques in gastroenterological endoscopy. All papers include a high-quality video and are published with a Creative Commons CC-BY license. Endoscopy E-Videos qualify for HINARI discounts and waivers and eligibility is automatically checked during the submission process. We grant 100% waivers to articles whose corresponding authors are based in Group A countries and 50% waivers to those who are based in Group B countries as classified by Research4Life (see: https://www.research4life.org/access/eligibility/ ). This section has its own submission website at https://mc.manuscriptcentral.com/e-videos .

## References

[1] Hatta W Tsuji Y Yoshio T Prediction model of bleeding after endoscopic submucosal dissection for early gastric cancer: BEST-J score Gut 2021 70 476 484 10.1136/gutjnl-2019-319926 32499390 PMC7873424

[2] Maekawa S Nomura R Murase T Complete closure of artificial gastric ulcer after endoscopic submucosal dissection by combined use of a single over-the-scope clip and through-the-scope clips (with videos) Surg Endosc 2015 29 500 504 25052125 10.1007/s00464-014-3725-1 PMC4293458

[3] Choi KD Jung HY Lee GH Application of metal hemoclips for closure of endoscopic mucosal resection-induced ulcers of the stomach to prevent delayed bleeding Surg Endosc 2008 22 1882 1886 10.1007/s00464-008-9743-0 18270775

[4] Inada T Sumida Y Homma H Novel clip method for endoscopic submucosal dissection defect closure reducing submucosal dead space in antithrombotic gastric patients Endoscopy 2024 56 E45 E46 10.1055/a-2223-4475 38232769 PMC10794086

[5] Nishizawa T Banno S Kinoshita S Feasibility of endoscopic mucosa-submucosa clip closure method (with video) Endosc Int Open 2018 6 E1070 E1074 30105296 10.1055/a-0630-0566 PMC6086683

